# James Truman, D.D.S., L.L.D.

**Published:** 1915-01

**Authors:** 


JAMES TRUMAN, D.D.S., L.L.D.—Dr. Truman died at home in Philadelphia, November 26, 1914, at the age of 88. Dr. Truman was born in Abington, Pa., November 22, 1826. He graduated from the Pennsylvania College of Dental Surgery in 1854. The honorary degree of L.L.D. was conferred upon him by the University of Pennsylvania in 1904.

Dr. Truman practiced dentistry, taught in dental colleges and edited dental journals almost from the time of his graduation. He began teaching in the Pennsylvania College of Dental Surgery in 1864 and continued until 1876, when he was made professor in the Dental Department of the University of Pennsylvania, and was Dean of this school from 1883 to 1895, when he was retired on the Carnegie Foundation Pension List as an Emeritus Professor.

As an editor he began his work with the *Dental Times* in 1865 and continued to 1869. He edited the *International Dental Journal* from 1890 to 1905, when it ceased to exist.

He was a forceful writer, because he was an earnest thinker. He was careful and painstaking and whatever he wrote he conscientiously believed. Sometimes his statements were considered radical and were not universally accepted, but even those who did not agree with him were compelled to believe in the honesty of his convictions, and could not help admiring the courage of his statements. He was a man of the highest professional conceptions and ideals, and had all the determination of a Scotchman to compel him to declare his convictions, even though it sometimes brought him into antagonism with his friends. He had a scientific mind and had no patience with any proposition that was not thoroughly grounded in fact. He wrote much, and all of his writings are worth preserving, as he was a close observer and had the capacity for recording in attractive manner his observations. His editorial work stands for the best that has ever graced dental journalism, perhaps not so brilliant as some, but always of high literary merit and technically sound.

In dental society work he was a giant, among the greatest of his time. It was a rare treat for us, as young men, to witness a debate between him and such men as W. H. Morgan, J. Taft, and others of their time.

As an educator we are not so familiar with his personal work, but we have always understood that he was a successful leader and made a deep impression on the thinking of his students. He was one of profession's greatest men. He made his own mark on many aspects of professional life, not so much in the technical, as in the scientific and literary departments. Because of his strong convictions and positive methods of expressing his convictions, and possibly because of a lack of social approach he was not a popular idol; but to those who knew him intimately he was most highly esteemed. He kept his mental faculties to the last and continued his interest in his beloved profession even though he had severed all active participation with it.

